# Bad air, amulets and mosquitoes: 2,000 years of changing perspectives on malaria

**DOI:** 10.1186/1475-2875-12-232

**Published:** 2013-07-09

**Authors:** Ernst Hempelmann, Kristine Krafts

**Affiliations:** 1 Department of Pharmacology, University of Witwatersrand, Johannesburg, South Africa and Dorfhalde 14, D-88662, Überlingen, Germany; 2 Department of Oral Pathology, University of Minnesota School of Dentistry, 515 Delaware Street SE, Minneapolis, MN 55455, USA

## Abstract

For many centuries, scientists have debated the cause and best treatment of the disease now known as malaria. Two theories regarding malaria transmission – that of “bad air” and that of insect vectors – have been widely accepted at different times throughout history. Treatments and cures have varied accordingly over time. This paper traces the evolution of scientific consensus on malaria aetiology, transmission, and treatment from ancient times to the present day.

## Background

There are certain diseases which have been historically shrouded in mystery, their causes attributed to magical or spiritual forces. Ovarian teratoma, for example, a tumour named after the Greek τέρας (monster), is a neoplasm composed of hair, teeth, skin and other mature tissues. Aristotle attributed teratomas to hair that had been swallowed by the patient and deposited in various body tissues. But other theorists claimed more ominous origins: teratomas were variously reported to be a consequence of sexual relations with the devil, an expression of a nightmare (incubus), evidence of engagement in witchcraft, or a punishment for wickedness.

Malaria has long been a member of this fascinating group of diseases with veiled origins. For many centuries it was believed that certain diseases, such as malaria and cholera, were caused by miasma (μíασμα, ancient Greek: pollution, defilement), a poisonous vapour or mist filled with particles from decomposed matter (miasmata). Prior to the introduction of the microscope, everything floating above ground that was invisible to the human eye, including dust particles and bacteria, was called “air” – so in a sense, perhaps this early aetiological speculation was not far from the truth.

### Hippocrates

In 400 BCE, Hippocrates discussed the aetiology of selected diseases in his treatise “On Airs, Waters, and Places” [1]. In ancient times, long before the term malaria was coined, the disease was described variously as “marsh fevers”, “agues” (from the Latin *febris acuta*), “tertian fevers”, “quartan fevers”, or “intermittent fevers.” Most terms originated from the writing of Hippocrates, who described the unhealthiness of the air in certain environments as it related to fatal diseases with quartan fevers:

*This disease is habitual to them both in summer and in winter, and in addition they are very subject to dropsies of a most fatal character; and in summer dysenteries, diarrhoeas, and protracted quartan fevers frequently seize them, and these diseases when prolonged dispose such constitutions to dropsies, and thus prove fatal*[1].

### Quintus Serenus Sammonicus

**Figure 1:**
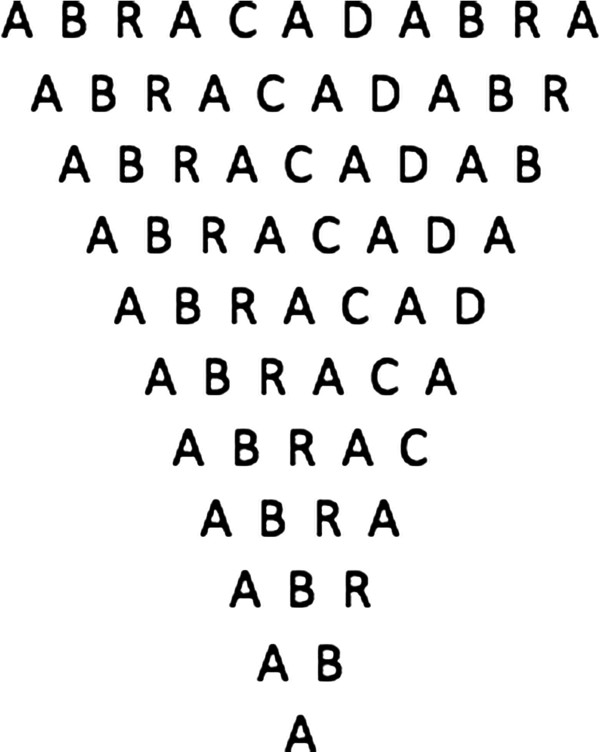
Sammonicus’ anti-pyretic abracadabra talisman.

The somewhat mystical concept of bad air set the stage for an alchemistic malaria treatment in the third century CE. Quintus Serenus Sammonicus, physician to the Roman emperor Caracalla, directed patients suffering from fever and ague to wear an amulet with the inscription “abracadabra” (Figure 1) in his didactic medical poem “Liber Medicinalis:”

Inscribis chartae, quod dicitur Abracadabra,

Saepius: et subter repetas, sed detrahe summae,

Et magis atque magis desint elementa figuris

Singula, quae semper rapies et coetera figes,

Donec in angustam redigatur litera conum.

*His lino nexis collum redimire memento*[2].

*Write several times on a piece of paper the word ‘Abracadabra,’ and repeat the word in the lines below, but take away letters from the complete word and let the letters fall away one at a time in each succeeding line. Take these away ever, but keep the rest until the writing is reduced to a narrow cone. Remember to tie these papers with flax and bind them round the neck*[3].

After wearing the talisman for nine days, it was to be thrown over the shoulder into an eastward-running stream. Failing this treatment, Sammonicus recommended the application of lion’s fat, or the wearing of cat’s skin tied with yellow coral and green emeralds around the neck [3].

Some scholars dismiss the word abracadabra as meaningless. Others, however, translate it as, “let the thing be destroyed”, “Out, bad spirit, out” (from the Hebrew words *Abrai seda brai*), or “Father, Holy Ghost, Word” (from the Hebrew words *Ab, Ruach, Dabar*) [3,4].

### Marcus Terentius Varro

The “bad air” or miasma theory of malaria aetiology remained an accepted hypothesis well into the 19th Century CE, when mosquitoes were found to be the transmitting agent of the disease. However, the correlation between disease and insects has its roots in antiquity.

The avoidance of insects is certainly not a new phenomenon. In the 26th Century BCE, the Egyptians ate foods of the onion family – most likely garlic – to ward off mosquitoes. Herodotus, a Greek historian who lived in the Fifth Century BCE, described the practice:

*On the pyramid it is declared in Egyptian writing how much was spent on radishes and onions and leeks for the workmen, and if I rightly remember that which the interpreter said in reading to me this inscription, a sum of 1,600 talents of silver was spent*[5].

Insects have been recognized as agents of death and decay since ancient times. In the Old Testament of The Bible, the fourth plague of Egypt consisted of swarms of flies (עָרוֹב):

Else, if thou wilt not let my people go, behold, I will send swarms of flies upon thee, and upon thy servants, and upon thy people, and into thy houses: and the houses of the Egyptians shall be full of swarms of flies, and also the ground whereon they are.

Exodus 8:21 (King James Bible, Cambridge Ed)

The ancient Hebrew word, עָרוֹב , often translated simply as “flies”, most likely represents a multitude of various sorts of insects – not only flies, but gnats, wasps, and hornets – of a more pernicious nature than their common counterparts.

Years later, disease began to be associated not only with insects but with tiny organisms too small to be seen. Marcus Terentius Varro (116–27 BCE), a Roman scholar and writer, recognized the importance of tiny creatures in the pathogenesis of disease:

*Advertendum etiam, siqua erunt loca palustria, et propter easdem causas, et quod crescunt animalia quaedam minuta, quae non possunt oculi consequi, et per aera intus in corpus per os ac nares perveniunt atque efficiunt difficilis morbos. Fundanius, Quid potero, inquit, facere, si istius modi mi fundus hereditati obvenerit, quo minus pestilentia noceat? Istuc vel ego possum respondere, inquit Agrius; vendas, quot assibus possis, aut si nequeas, relinquas*.

*Precautions must also be taken in the neighbourhood of swamps, both for the reasons given, and because there are bred certain minute creatures which cannot be seen by the eyes, which float in the air and enter the body through the mouth and nose and there cause serious diseases. “What can I do”, asked Fundanius, “to prevent disease if I should inherit a farm of that kind?” “Even I can answer that question”, replied Agrius; “sell it for the highest cash price; or if you can't sell it, abandon it”*[6].

Over 1,500 years passed before the term “malaria” came into use. The word malaria has its roots in the miasma theory, as described by historian and chancellor of Florence Leonardo Bruni in his *Historia Florentina*, which was the first major example of Renaissance historical writing:

*Recepto Florentini castello munitissimo praesidioque imposito, quid iam agendum foret consultabant. Erant quibus optimum videretur exercitum reducere,praesertim morbis***gravitateque coeli***laborantem, et longa difficilique militia per aestatis autumnique ferventissimos ardores insalubribus locis confectum, missione etiam multorum a duce concessa diminutum: nam postquam diutius in his locis commoratum est, multi, vel tedio castrorum vel metu valetudinis adversae, commeatum a duce postulaverant*[7].

*Avuto i Fiorentini questo fortissimo castello e fornitolo di buone guardie, consigliavano fra loro medesimi fosse da fare. Erano alcuni a' quali pareva sommamente utile e necessario a ridurre lo esercito, e massimamente essendo affaticato per la infermità e per la ***mala aria***e per lungo e difficile campeggiare nel tempo dell'autunno e in luoghi infermi, e vedendo ancora ch'egli era diminuito assai per la licenza conceduta a molti pel capitano di potersi partire: perocchè, nel tempo che eglino erano stati lungamente a quello assedio, molti, o per disagio del campo o per paura d'infermità, avevano domandato e ottenuto licenza da lui*[8]*.*

*After the Florentines had conquered this stronghold, after putting good guardians on it they were discussing among themselves how to proceed. For some of them it appeared most useful and necessary to reduce the army, more so as it was extremely stressed by disease and mala aria (bad air), and due to the long-lasting and difficult camps in unhealthy places during the autumn. They (the Florentines) further considered that the army was reduced in numbers due to the leave permits granted to many soldiers by the officers. In fact during the siege many soldiers had asked and obtained the permit to leave due to the camp hardships and fear of illness.* (Translation into English by Paolo Arese, personal communication)

The Italian term “mal'aria” (bad air) was introduced into England 300 years later by Horace Walpole in a letter he wrote on 5 July, 1740: *“There is a horrid thing called the malaria, that comes to Rome every summer, and kills one.”*[9]. John MacCulloch introduced the word into the English scientific literature in 1827 [9]. However, Charles Laveran, the first to see the malarial organism in blood in 1893, intensely disliked the name malaria. He considered the term unscientific and vulgar, preferring the name “paludisme” (Latin: palus = swamp) which is still used in France today [10]. Currently, the use of the word malaria is restricted to the disease and its symptoms (and not its causative agent).

Perhaps in part due to the name of the disease, the aetiological concept of bad air prevailed until the latter part of the 19th Century. During his many travels, the journalist and African explorer Henry Morton Stanley (1857–1932) erected a glass screen on his boat, which he used for his trips on the Congo River, as protection against miasma [11].

### Joannes Maria Lancisius

Appointed as physician to three Popes (Innocent XI, Innocent XII and Clement XI), Joannes Lancisius was one of the greatest physicians of his time. In his book De noxiis paludum effluviis, eorumque remediis (“On the noxious emanations of swamps, and their remedies”), he describes the transfer of deadly diseases by animals, noting that *“Venenata animalia non occidunt vulnere, sed infuso per vulnus venetico liquido”* (Venomous animals do not kill by injuries, but they inject a poisonous liquid through the wound) [12].

Lancisius strongly advocated the use of Cortex Peruvianus (Peruvian bark) for the treatment of periodic fever. Extracts of this bark had been used to treat malarial fever since the early 1600 s. However, the use of fine powder of Peruvian bark, mixed into French wine, was not without problems, and many physicians were not in favour of the use of the bark. In 1707, Anthony van Leeuwenhoek wrote to Heer van Wikhuysen, *“This medicine is not to be used but with the utmost caution, for that otherwise it may be so prejudicial to the body, that tho the fever should be removed, the subsequent inconveniences may be worse than the disease itself''*[13].

Neither of the eminent Italian physicians Giovanni Lancisi nor Francesco Torti used the term malaria. It remained a term used exclusively in Italian folk medicine.

### Francesco Torti

**Figure 2:**
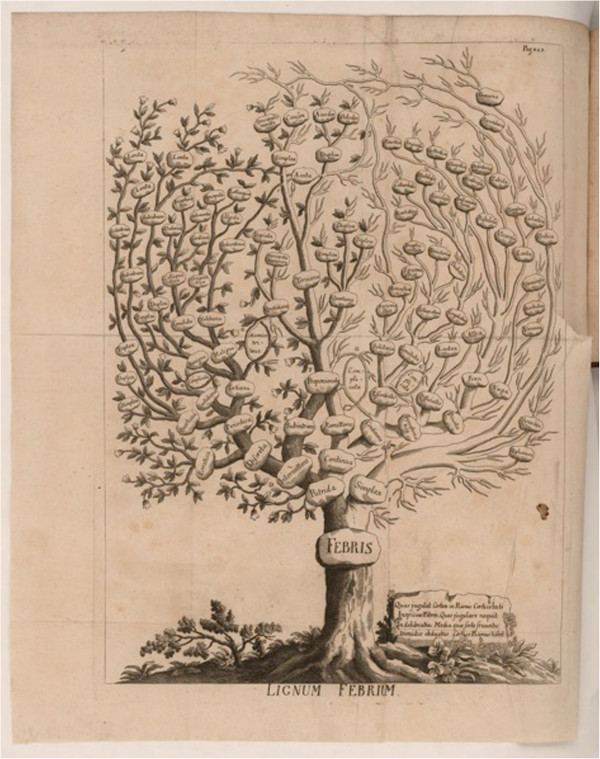
**Torti Fever tree in the shape of a stylized *cinchona *plant.** (Courtesy The John Carter Brown Library at Brown University).

In 1756, Francesco Torti defined a new standard of care for the use of Peruvian bark. Using a drawing of the Lignum Febrium tree, Torti used bark-covered branches to represent conditions for which *cinchona* was effective. Barkless, leafless branches were used to represent conditions for which *cinchona* was ineffective (Figure 2). His elegantly illustrated publication showed that only intermittent fevers were responsive to treatment with *cinchona* bark [14].

### Carl Linnaeus

Malaria has long been distinguished from other fevers in two aspects. First, malaria is associated with a unique and characteristic periodic fever, and second, only malaria is curable by quinine.

In 1735, Swedish botanist Carl Linnæus travelled to the University of Harderwijk in Holland where he earned his medical degree. His graduate thesis was entitled, “Inaugural thesis in medicine, in which a new hypothesis on the cause of intermittent fevers is presented. By the favour of God, three times the best and the greatest, submitted by Carolus Linnæus from Småland, Sweden, a Wredian scholar” [15].

In his 24-page, 84-chapter thesis, Linnæus compared different regions of Sweden and found clay and intermittent fevers to be geographically connected. He concluded that very small clay particles were responsible for the symptoms of the disease, and proposed that intermittent fevers originated in – and only in – places with clay-rich soil:

*24. Frigus non est causa vera, quia: Rarissima est Febrium intermittentium in frigidissimis terrae.* (Cold is not the true cause, since intermittent fever is very rare in the coldest parts of the country.)

*38. α. Argilla in Uplandia* &*quidem circa Stockholmiam Upsaliamque, ut* &*in campis Scaniæ frequentissima est, ubi etiam febres intermitt. fræquentissimæ.* (α. Clay is very common in Uppland, around Stockholm and Uppsala as well as in the fields of Skåne, where intermittent fevers are most frequent.)

Further in his thesis, he wrote:

*77. α.* China *de qua medici omnes videndi; Tinct. Chin*æ *fuit arcanum maxime exclamatum. γ.* Artemisia, Centaurium & Gentiana*, nec non* Nux vomica*, quatenus amara, agunt.* (α. *Quinine* is known to all physicians. Tinct. Chinæ is a very important and trustworthy remedy. γ. *Artemisia, Centaurium* &*Gentiana*, but not *Strychnos nux vomica*, because they are bitter, are effective.)

**Figure 3:**
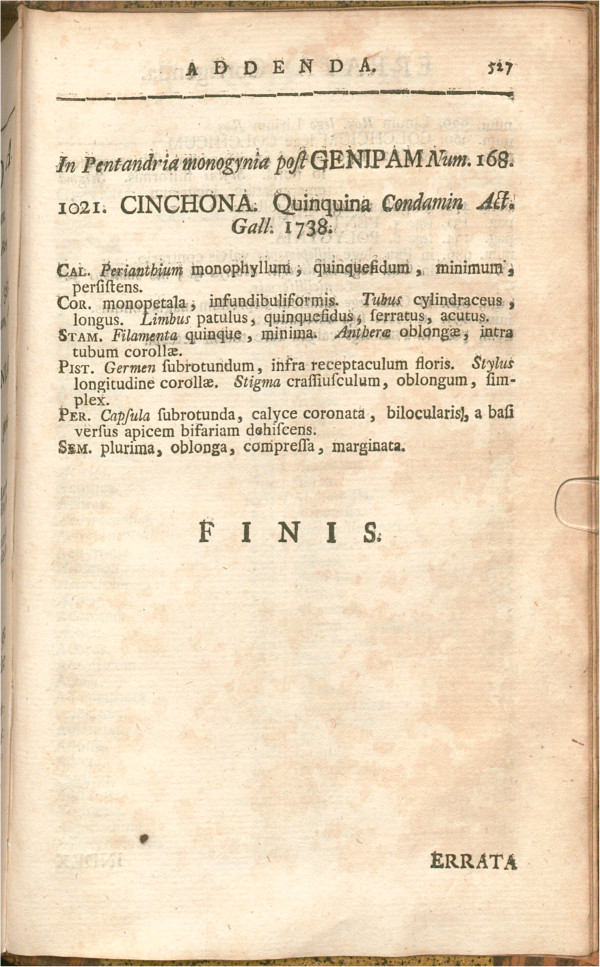
**Linnæus’ listing of the *Cinchona *genus.** (Courtesy Bayerische Staatsbibliothek, München).

The drug quinine is extracted from the bark of a tree now known as the *Cinchona* tree [16]. In 1742, Linnæus named the genus of this tree in his seminal textbook *Genera Plantarum*[17]. In the second revised and enlarged edition, 1,021 plant genera were listed, including, as the last entry, the newly described *Cinchona* genus (Figure 3).

### Albert Freeman Africanus King

In 1851, in his address to the Medical Society of North Carolina, Charles E Johnson refuted the doctrine of the miasmatic origin of malaria. As evidence, he pointed to the “sickly” constitution of the residents of Gibraltar, a town built on a bed of dry red sand with no ponds or marshes to furnish the decomposing vegetable matter necessary for the generation of miasma. In addition, he noted that no chemical analysis or microscopic investigation had ever been able to identify miasma, and that air composition was always found to be 78 parts nitrogen, 21 parts oxygen, and one part carbonic acid [18].

By 1883, the American physician, Albert Freeman Africanus King, had assembled 19 facts in support of the mosquito as the origin of malarial disease [19]:

1 Malaria is most common in areas (swamps, fens, jungles, marshes, etc.) where mosquitoes are endemic;

2 Malaria is most common at temperatures conducive to mosquito growth;

3 Malaria does not occur at cold temperatures;

4 Malaria is most common in equatorial and coastal regions;

5 Malaria occurs frequently in areas of dense foliage;

6 Forests may obstruct malaria transmission;

7 Malaria may spread to areas that are miles away;

8 Malaria may spread to previously unaffected places when soil is excavated;

9 A large body of water may prevent the spread of malaria;

10 Previously malarial countries, when cleared up, may become free of disease;

11 The threat of malaria is greatest near the surface of the earth;

12 Malaria transmission is greatest during the night;

13 The danger of acquiring malaria is greater after sleeping in the night air;

14 Fire protects against malaria;

15 City air has a protective effect against malaria;

16 Malaria is most prevalent in later summer and fall;

17 Malaria is arrested by canvas curtains, gauze veils, and mosquito nets;

18 Malaria affects infants much less frequently than adults; and

19 Of all human races the white is most susceptible to marsh-fevers, the black least so;

### Johann Heinrich Meckel

Few families have had such an impact on medicine as the Meckel family, which for four generations contributed greatly to anatomy, pathology and the biological sciences. The last member of the Meckel lineage, Johann Heinrich Meckel (1821–1856), took the instructor’s position in pathologic anatomy at the University of Berlin that his great-grandfather had held at the Charité. After his untimely death from pulmonary disease, Meckel’s position was filled by Rudolf Virchow [20].

In the 1800s, malaria was endemic in all of Central Europe, except Lichtenstein. It was well known that patients who died of malaria had black deposits in their organs. According to Hippocrates, these black deposits were characteristic of malaria, and were attributable to bile. This view prevailed until Johann Meckel proved otherwise.

In 1846, a patient named Adelheid B died at the age of 43 after 24 years in various hospitals for mentally ill patients. Malaria had not been diagnosed prior to her death. Heinrich Meckel conducted her autopsy. He found her brain to be dark brown, with all capillaries filled with brownish particles which were without Brownian motion. The spleen was enlarged and likewise dark brown, with similarly filled capillaries. Meckel concluded that the brown pigment was a blood product (“*aus dem Blutroth entsteht ein schwarzes Pigment*”) [21].

**Figure 4:**
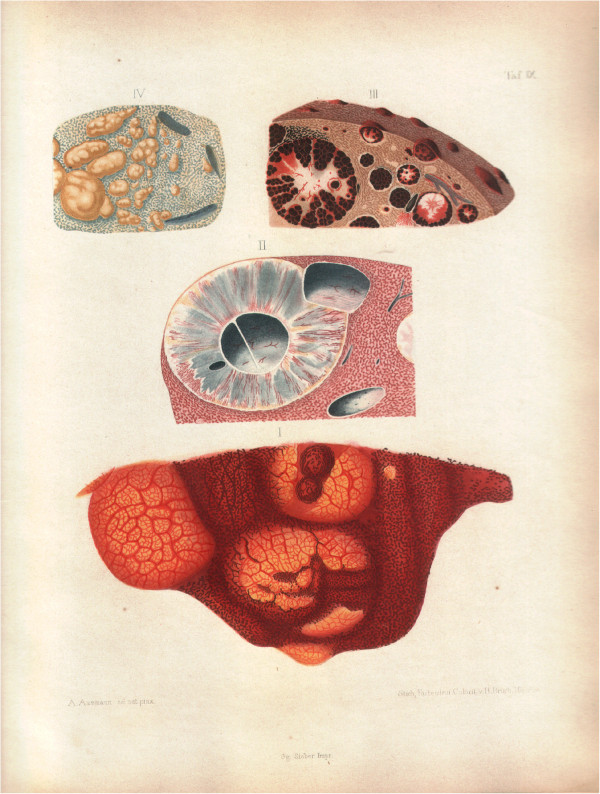
**Frerichs’ drawings of the deposition of pigment in internal organs of intermittens patients.** (Courtesy Staatsbibliothek zu Berlin – Preussischer Kulturbesitz).

Meckel did not associate the pigment with malaria. But only a few years later the causal relationship of this brown pigment to malaria was established by Virchow and Frerichs, and malaria was recognized to be a disease of the blood (Figure 4) [22,23]. Meckel had erroneously assumed the pigment was chemically identical to melanin, but Virchow correctly associated the pigment with haematin crystals (“*aus Hämatin condensiert sich allmählich körniges oder krystallinisches Pigment*”).

It is now known that this brown pigment is formed during the digestion of haemoglobin and produced by the malaria parasite through biocrystallization [24]. The presence of brown pigment (also called haemozoin) in organs at autopsy is a strong indicator of malarial infection.

### Charles Louis Alphonse Laveran

In the early years of microscopy, staining technology was in its infancy. The French army doctor, Laveran, had to use unfixed and unstained blood for his experiments, and could see in the blood of his patients a dark pigment that was, according to Virchow, the result of infection with malaria. Some years passed before Malachowski developed a stain which allowed for differential identification of blood parasites [25].

Laveran agreed with Meckel with regard to the brown pigment, maintaining that the pigment was chemically related to melanin: “*Melan*æ*mia is specially very pronounced in individuals who died from acute paludisme (pernicious attacks); the colour which it gives to certain organs, particularly to the spleen, the liver, and the grey substance of the brain, is almost always sufficient to show from microscopic examination if death is the result of paludisme”*[26].

**Figure 5:**
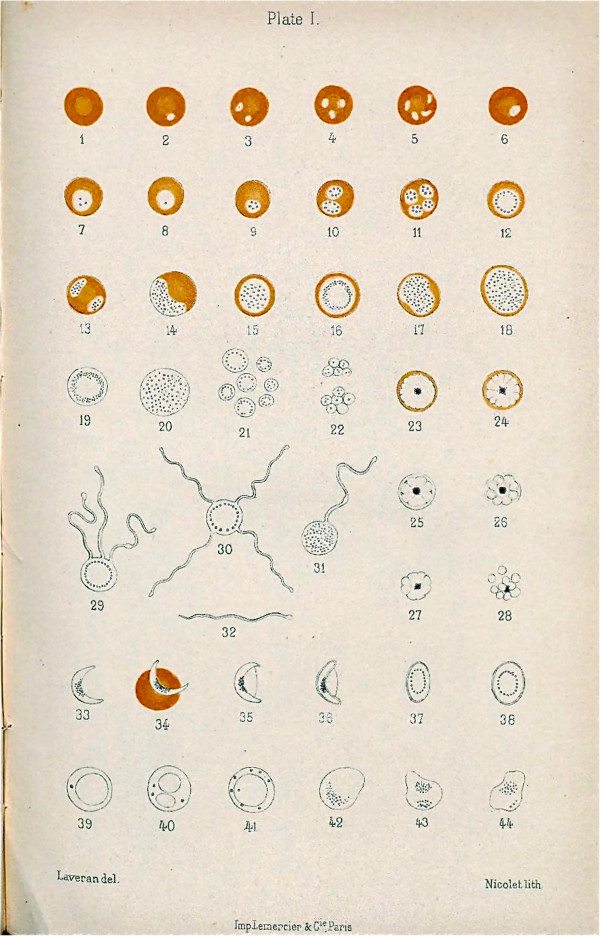
Laveran’s drawing of malaria parasites.

More convincing was his observation of male gametocytes undergoing exflagellation (Figure 5). In 1880 at the Military Hospital at Constantine in Algeria he discovered, on the edges of pigmented spherical bodies in the blood of a patient suffering from malaria, filiform elements resembling flagella which were moving very rapidly: “*I was still hesitating whether these elements were parasites, when on November 6th, 1880, on examining the pigmented spherical bodies mentioned above, I observed, on the edge of several of these elements, moveable filaments or flagella, whose extremely rapid and varied movements left no doubt as to their nature”*[26].

### Ronald Ross

**Figure 6:**
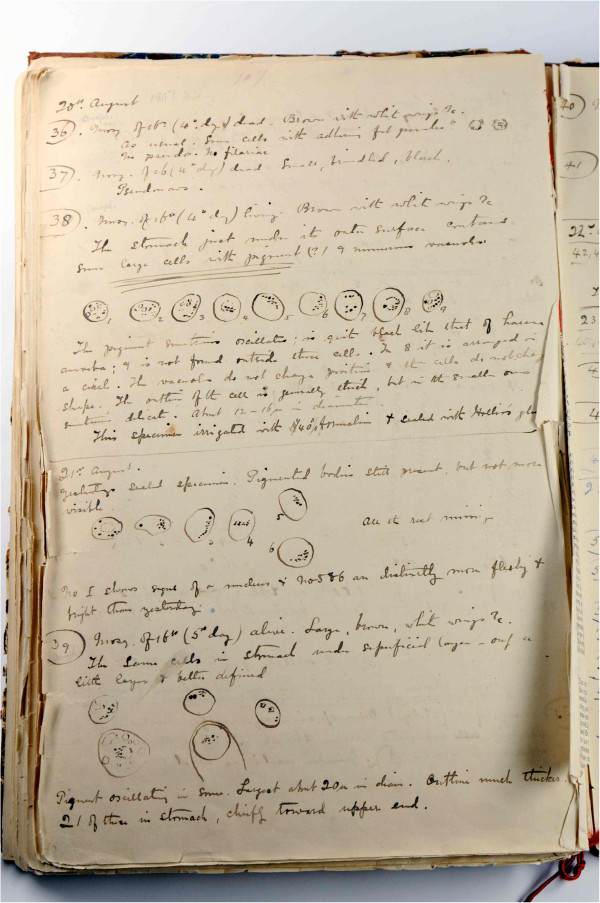
**Ross’ Diary and Notes of Researches on Malaria, Book I, page 107.** (Courtesy Archives Service, London School of Hygiene & Tropical Medicine).

The first person to provide definitive proof that mosquitoes carried malaria was British army surgeon, Ronald Ross. Working in Secunderabad, India, under the tutelage of his mentor, Patrick Manson, Ross undertook a meticulous, two-year search, microscopically examining thousands of brindled grey and white mosquitoes fed with malarial blood, looking for a pathogen inside the mosquito. In 1897, he obtained a few mosquitoes which belonged to a species with spotted wings. Ross fed these mosquitoes with blood from a patient named Husein Khan, whose blood contained numerous crescent-shaped cells (Figure 6) [27]. It was in these spotted mosquitoes, now known to be *Anopheles* species, that Ross detected characteristic pigmented bodies in the stomach wall. Because mosquitoes do not produce pigment (haemozoin), Ross deduced that the pigment was causally related to malaria [27].

### Robert Koch

**Figure 7:**
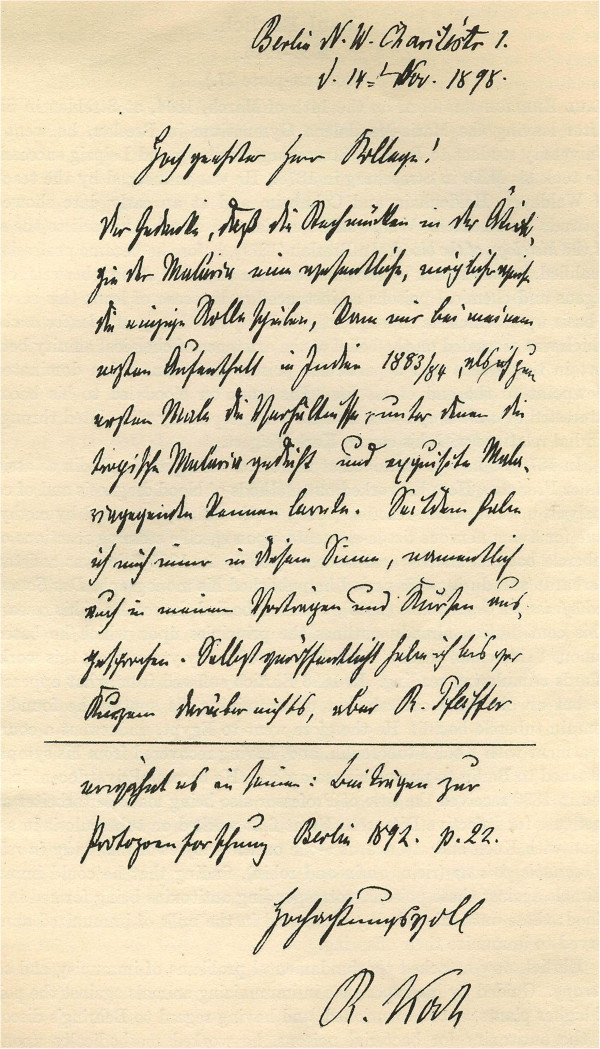
Letter from R Koch to G Nuttall.

Koch, whose prime quest was tuberculosis control, began malaria fieldwork in 1897. In his African studies he found that in some malaria-ridden villages, all children had malaria and splenomegaly, but as the children grew, the splenomegaly disappeared, and their blood no longer contained the parasites in demonstrable numbers. Eventually, the children became immune to malaria. He favoured the mosquito-borne theory based on concepts he developed during a visit to India in 1883. Koch describes the origins of his theory briefly in a letter he wrote to his Cambridge colleague GHF Nuttall in 1898 (Figure 7) [28]:

Berlin Northwest Charitéstr 1

d. 14ten Nov. 1898

Hochgeehrter Herr Kollege!

Der Gedanke, dass die Stechmücke in der Ätiologie der Malaria eine wesentliche, möglicherweise die einzige Rolle spielt, kam mir bei meinem ersten Aufenthalt in Indien 1883/84, als ich zum ersten Mal die Verhältnisse, unter denen die tropische Malaria gedeiht, und exquisite Malariagegende kennen lernte. Seitdem habe ich mich immer in diesem Sinn, namentlich auch in meinen Vorträgen und Kursen, ausgesprochen. Selbst veröffentlicht habe ich bis vor kurzem darüber nichts, aber R. Pfeiffer erwähnt es in seinem: Beiträge zur Protozoenforschung Berlin 1892, S. 22.

HochachtungsvollR Koch

Berlin Northwest Charitéstr 1

November 14th, 1898

Highly honoured Colleague!

The idea that mosquitoes play an important, possiblythe only part in the aetiology of malaria, came to meduring my first sojourn in India in 1883/84, when forthe first time I came to know the conditions underwhich tropical malaria flourishes and came to knowexquisite malarious regions. Since then, I have alwaysexpressed this view, especially in my lectures andcourses. Until recently I have not published anythingabout this subject, but R. Pfeiffer mentions it in his: Beiträge zur Protozoenforschung, Berlin, 1892, S. 22.

HochachtungsvollR Koch

### Giovanni Battista Grassi

Giovanni Battista Grassi, an Italian zoologist, scientifically distinguished different species of mosquitoes. In 1898, he unequivocally identified *Anopheles claviger* (syn *A. maculipennis*) (Greek *anofelís*: good-for-nothing) as the sole vector of malaria in Italy [29].

### Patrick Manson

Patrick Manson, a Scottish physician, realized that the exflagellation described by Laveran could not take place within the bloodstream, but only when the parasite was outside of the human body, exposed to moisture and a lower temperature, such as within the stomach of some insects. Manson’s malaria theory consisted of two parts: the metamorphosis of the “flagella” occurs in the mosquito’s stomach and the liberated flagella enters drinking water. The second part of his theory – which stated that drinking contaminated water was the reason for getting malaria – turned out to be completely wrong [27].

In 1900, Manson provided convincing, if disturbing, experimental proof of the role played by mosquitoes in the propagation of malarial fevers. On 29 September, Manson reported a positive infection experiment using *Anopheles* mosquitoes imported from Rome. The journey from Rome to London lasted about three and a half days, and most of the mosquitoes arrived in London alive and in good condition. The insects were permitted to bite the fingers and hands of Manson’s healthy, 23-year-old son, Patrick Thurburn Manson, who had never suffered from malaria, and who had not been abroad since the age of three. He passed through a sharp attack of double benign tertian fever malaria about 14 days later. Quinine was administered and the young Manson soon returned to good health [30].

According to Manson, these experiments plainly indicated that the practical solution of the malaria problem lay in:

1 Avoiding the neighbourhood of native houses, the perennial source of malaria parasites;

2 The destruction, so far as practicable, of *Anopheles'* breeding pools; and

3 Principally: protection from mosquito bites.

### Camillo Golgi

**Figure 8:**
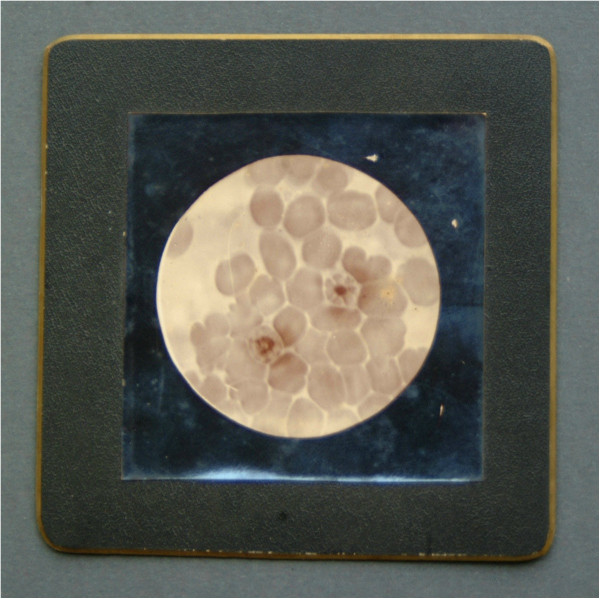
**Golgi’s original microphotograph of a daisy-like malaria blood preparation.** (Courtesy Museum for the History of the University of Pavia).

Between 1885 and 1892, Bartolomeo Camillo Golgi studied the asexual cycle of the malaria parasite and related its stages to the observed stages of the various forms of malaria (Figure 8). He found that the febrile bouts coincided with segmentation (“Golgi’s law”). His work was the first to demonstrate the concept of a biological clock.

In studying the relationship between the biological cycle of the malarial parasite and the onset of fever, Golgi found that the two types of intermittent malarial fevers (tertian, occurring every other day, and quartan, occurring every third day) were caused by different species of *Plasmodium* and that the paroxysms of fever coincided with the rupture and release of merozoites into the bloodstream [31].

### Fritz Schaudinn

In 1903, Fritz Schaudinn reported that the female *Anopheles* mosquito injected sporozoites into the bloodstream, after which the sporozoites directly entered red blood cells. Schaudinn had been struggling for some time to solve the mystery of the mode of entrance into the body by malaria parasites. From 1901–1904, he worked at the malaria station in Rovigno, a small town in Dalmatia (now Rovinj, Croatia), which was notorious for its high rate of malarial infection. He performed a set of five experiments in which he allowed mosquitoes to feed on his maid, who had *Plasmodium vivax* crescents in her blood, and subsequently used his own blood for invasion experiments.

**Figure 9:**
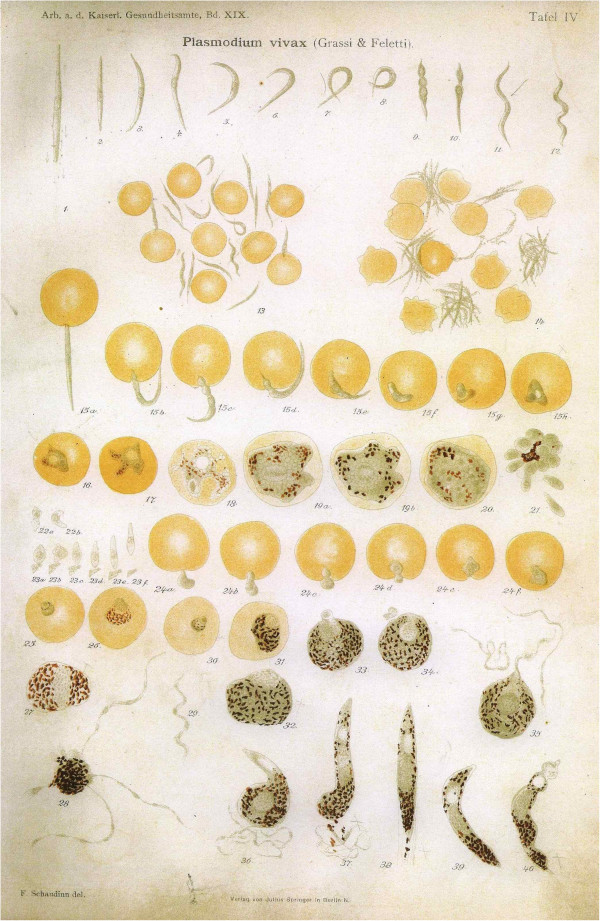
**Schaudinn’s drawings showing direct entry of erythrocytes by infective sporozoites in cells numbered 15 a–h.** (Courtesy Tropeninstitut Hamburg).

In the first four experiments, he failed to find any evidence of direct red cell invasion. In the last experiment, however, he observed the malarial sporozoite (the end-stage form in the sexual cycle of the parasite) directly entering a red cell. The sporozoites did not develop further within the red cell (Figure 9) [32]. No appropriate controls were used, and the experiment was not replicated. Unfortunately, Schaudinn died on 22 June, 1906 at the age of 34. Due to his impressive list of accomplishments, Schaudinn’s report of direct penetration of erythrocytes by infective sporozoites of *P. vivax* dominated scientific opinion until 1947, when Henry Shortt and Cyril Garnham showed that a phase of division in the liver preceded the development of parasites in the blood [33].

## Conclusion

Malaria remains one of the most important causes of human morbidity and mortality worldwide, with a tremendous impact in the developing world. Theories on the cause and transmission of malaria have evolved over time, from Hippocrates’ theory of bad air in the Fifth Century BCE to our current understanding of Plasmodial organisms as the causative agent of the disease. Treatments for malaria have likewise changed over time, from Sammonicus’ magical amulets in the Third Century CE to today’s anti-malarial drugs. Current anti-malarial treatments are much more sophisticated than ancient therapies. However, as some of the drugs in the anti-malarial arsenal are losing effectiveness, perhaps over time there may be a resurgence of bygone therapies.

## Competing interests

The authors have declared that they have no competing interests.

## Authors’ contributions

Both authors contributed to the research, drafting and editing of the paper. Both authors read and approved the final manuscript.
